# Cyclooxygenase 2 expression in pterygium

**Published:** 2007-04-27

**Authors:** Chun-Chi Chiang, Ya-Wen Cheng, Chien-Lin Lin, Huei Lee, Fuu-Jen Tsai, Sung-Huei Tseng, Yi-Yu Tsai

**Affiliations:** 1 Department of Ophthalmology, China Medical University Hospital, Taichung, Taiwan; 2 Institute of Medicine, Chung Shan Medical University, Taichung, Taiwan; 3 Department of Physical Medicine and Rehabilitation, China Medical University Hospital, Taichung, Taiwan; 4 Institute of Toxicology, Chung Shan Medical University, Taichung, Taiwan; 5 College of Chinese Medicine, China Medical University, Taichung, Taiwan; 6 Department of Medical Genetics, China Medical University Hospital, Taichung, Taiwan; 7 Department of Ophthalmology, National Cheng Kung University Hospital, Tainan, Taiwan

## Abstract

**Purpose:**

Following the recent discovery of an abnormal expression of the *p53* gene in the epithelium in pterygium, some researchers felt that pterygium is a tumor rather than a degenerative disease. Ultraviolet (UV) radiation has been reported to be associated with pterygium formation, however the mechanism whereby UV induces uncontrolled proliferation in pterygial cells is unclear. Because cyclooxygenase 2 (COX 2) was reported to exist and play an important role in UV-related cutaneous carcinogenesis, it is logical to suspect that COX 2 existed in pterygium. This study was designed to investigate the expression of COX 2 in pterygium.

**Methods:**

Immunohistochemical staining using a monoclonal antibody to COX 2 was performed on 90 pterygial specimens, 40 normal conjunctiva, and 5 normal limbus.

**Results:**

In the pterygium group, 75 (83.3%) specimens stained positive for COX 2. The staining was limited to the cytoplasm of the epithelial layer and predominantly over the basal epithelial layer. No substantial staining was visible in the subepithelial fibrovascular layers. All specimens were negative in the normal conjunctiva and limbus group.

**Conclusions:**

The present study showed COX 2 existed in pterygium. Given the role of COX 2 in cutaneous tumor carcinogenesis, we suggest COX 2 may also play a role in pterygium formation. This study could be used as the basis for future surveys of the causal relationship between COX 2 and pterygium as well as the effect of COX 2 inhibitor in preventing primary or recurrent pterygium.

## Introduction

Pterygium has long been considered to be a chronic degenerative condition. However, after overexpression of the p53 protein was found in the epithelium of pterygium, some researchers began to feel that pterygium was an ultraviolet (UV)-related tumor rather than a degenerative disease [1-5].

The mechanism by which UV light induces uncontrolled proliferation in pterygial cells is under investigation, but still remains unclear. The noxious effects of UV irradiation are moderated either directly by the UV phototoxic effect or indirectly by the formation of radical oxygen species (ROS) [6,7]. ROS are harmful to cells, because they injure cellular proteins, lipids, and DNA in a process known as oxidative stress [6,7]. Moreover, ROS can induce cyclooxygenase 2 (COX 2) formation via activation of the NF-kB signaling pathway [8]. Both ROS and COX 2 were found to play the most important role in UV-related cutaneous carcinogenesis [6-11].

If pterygium is a UV-related uncontrolled cell proliferation, it is logical to assume that ROS and COX 2 may be found in pterygium. Our previous research identified ROS and oxidative stress in pterygium [12]; however, there is no report about the presence of COX 2 in pterygium. If COX 2 is indeed expressed, it provides further evidence of UV-related uncontrolled cell proliferation.

To investigate whether COX 2 is present in pterygium, we set out to evaluate COX 2 expression in pterygium. In this study, COX 2 protein was studied immunohistochemically in both pterygium and normal conjunctiva and limbus.

## Methods

Informed consent was obtained from all individuals who participated in this study. Primary pterygium samples were harvested from 90 patients undergoing pterygium surgery. These were 51 males and 39 females, with an age range of 50-83 years and an average age of 64.2 years. Normal conjunctiva samples were collected from medial conjunctiva of 22 patients and superior conjunctiva of 18 patients without pterygium and pinguecula when they underwent cataract or vitreoretinal surgery. Five normal limbal specimens were collected from residual sclerocorneal rims in penetrating keratoplasty. The control group contained 26 males and 19 females, with an age range of 55-81 years and a mean of 68.3 years. This study was carried out with approval from the Human Study Committee of the China Medical University Hospital and National Cheng Kung University Hospital.

All specimens were fixed in formalin before being embedded in paraffin. Briefly, 3 μm sections were cut, mounted on glass, and dried overnight at 37 °C. All sections were then deparaffinized in xylene, rehydrated with alcohol, and washed in phosphate-buffered saline. This buffer was used for all subsequent washes. Sections for COX 2 detection were heated in a microwave oven twice for 5 min in citrate buffer (pH 6.0). Mouse anti-COX 2 monoclonal antibody (at a dilution of 1:200; Alexis Biochemicals, San Diego, CA) was used as the primary antibody The incubation time was 60 min at room temperature followed by a conventional streptavidin peroxidase method (LSAB Kit K675; DAKO, Glostrup, Denmark). Signals were developed with 3, 3'-diaminobenzidine for 5 min and counter-stained with hematoxylin. Negative controls were obtained by leaving out primary antibody. COX 2 protein expression in colon cancer tissue was used as positive control. Sections of paraffin-embedded colon cancer samples were collected from the Chung Shan Medical University Hospital (CSMUH) after obtaining written informed consent according to a biology study approved by the CSMUH Institutional Review Board. The histological diagnosis and clinic pathological staging were according to the WHO classification. The results were scored for the percentage of positive staining: score 0, no positive staining; score +, from 1% to 10%; score ++, from 11% - 50%; score +++, more than 50% positive cells. In this study, scores +, ++, and +++ were considered to be a positive immunostaining, and score 0 was seen as a negative immunostaining.

## Results

There were 51 males and 39 females in the pterygium group (age ranges from 50 to 83 years with an average of 64.2 years), and 26 males and 19 females in the control group (age range=55-81 years, mean=68.3 years).

**Figure 1:**
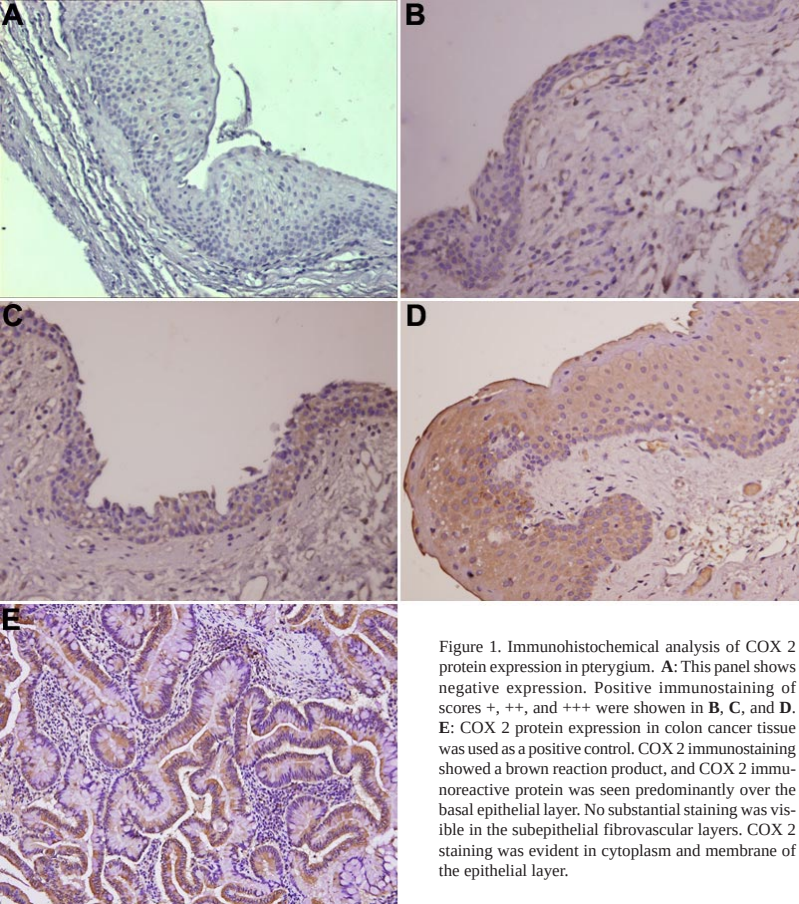
Immunohistochemical analysis of COX 2 protein expression in pterygium. **A**: This panel shows negative expression. Positive immunostaining of scores +, ++, and +++ were showen in **B**, **C**, and **D**. **E**: COX 2 protein expression in colon cancer tissue was used as a positive control. COX 2 immunostaining showed a brown reaction product, and COX 2 immunoreactive protein was seen predominantly over the basal epithelial layer. No substantial staining was visible in the subepithelial fibrovascular layers. COX 2 staining was evident in cytoplasm and membrane of the epithelial layer.

**Figure 2:**
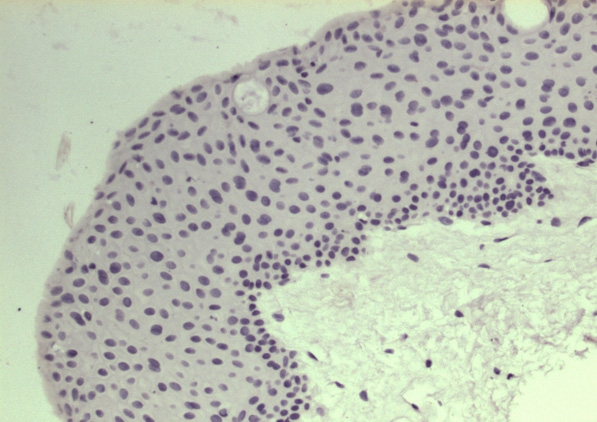
Normal conjunctiva showing negative immunostaining for COX 2. No substantial staining is visible in the epithelium and subepithelial fibrovascular layer of the normal conjunctiva.

In the pterygium group, the scores were as follows: 15 (16.7%) specimens were negative, 12 (13.3%) were +, 31 (34.4%) were ++, and 32 (35.6%) were +++. Seventy-five (83.3%) specimens were positive for COX 2 staining (Figure 1). The COX 2 staining was shown in cytoplasm and membrane of the epithelial layer and predominantly over the basal epithelial layer. No substantial staining was visible in the subepithelial fibrovascular layers. Goblet cells were intermingled in 10 of 90 pterygial specimens, and all were positive for COX 2 immunoreaction. There were no significant differences in sex and age between COX 2 positive and negative groups. In the normal conjunctiva and limbus groups, all specimens were negative for COX 2 staining (Figure 2).

## Discussion

There are two distinct forms of cyclooxygenase: COX 1 and COX 2. COX 1 is constitutively expressed in most tissues, whereas COX 2 is inducible by a variety of tumor-promoting agents, e.g. UV light [8-11,13]. A model has been proposed regarding the role of COX 2 in UV-related skin cancer: (1) it may serve to enhance prostaglandin E 2 production, which may function as a mitogen in an initiated cell population; (2) it may inhibit apoptosis and thus promote retention of UV-induced "sunburn cells," which are normally discarded by the epidermis as apoptotic cells; (3) it may alter the ability of the cells to attach to substrate and thus enhance their propensity to exhibit tumorigenic growth; and (4) it may enhance the formation of DNA adducts or decrease their repair [14,15]. Also, COX 2 was reported to be a key enzyme for inflammatory cytokine-induced angiogenesis, and the upregulation of vascular endothelial growth factor, basic fibroblast growth factor, and nitric oxide synthases, which are associated with tumor formation [16,17].

In our series, 83.3% of pterygial specimens had COX 2 expression while normal conjunctiva and limbus specimens had no COX 2 expression, indicating COX 2 indeed only existed in pterygium and not in normal conjunctiva and limbus. Moreover, the aforedescribed findings regarding the effects of COX 2 in cutaneous tumor formation have also been reported in pterygium, including disruption of apoptosis [3,18], limbal epithelial proliferation [19], abnormal p53 gene expression [1-5], and upregulation of basic fibroblast growth factor [20], vascular endothelial growth factor, and nitric oxide synthases [21]. Hence, we suggest that COX 2 may play a similar role in pterygium formation as that found in cutaneous tumorigenesis. Karim et al. [22] discovered COX 2 positive immunostaining in the cytoplasm and membranes in 96% (28 of 29 retinoblastoma patients) and in both differentiated and undifferentiated retinoblastomas, but not in normal portions. In our study, the similar staining pattern was also found. COX 2 had a positive immunoreaction in cytoplasm and membrane of pterygical tissues but not in normal conjunctiva and limbus. This may provide an indirect evidence that pterygium may be a tumor.

In cutaneous tumorigenesis, the mechanism involving COX 2 induced by UV irradiation was proposed to be via the generation of ROS, so there was a pathway of UV- ROS-COX 2 in cutaneous tumor [11]. In our previous survey, there was also oxidative DNA damage in pterygium, and lack of glutathione S-transferase M1, one of the antioxidant defense enzymes, which was found to be associated with early onset pterygium [12,23]. Hence, oxidative stress exists in pterygium. Our present study provides further evidence that there may be a similar pathway of UV-ROS-COX 2 in pterygium.

COX 2 is one of the key enzymes in the synthesis of prostaglandins. There is evidence to indicate that COX 2-induced prostaglandin synthesis contributes to UV-induced cutaneous tumorigenesis [9,10]. Hence, nonsteroidal antiinflammatory drugs (NSAIDs) are effective in skin cancer prevention [9,10]. If this relationship also exists in pterygium formation, COX 2 inhibitors and NSAIDs may be alternatives to mitomycin C, which has been widely used in pterygium but with several sight-threatening complications [24,25]. Further studies are necessary to evaluate the role of prostaglandins in pterygium formation and the effect of COX 2 inhibitors and NSAIDs in preventing primary and recurrent pterygium.

In conclusion, the present study shows that there is high expression of COX 2 in pterygium. Given the role of COX 2 in cutaneous tumorigenesis, we suggest that COX 2 may play a role in pterygium formation. This could serve as the basis of future surveys of the causal relationship between COX 2 and pterygium, the pathway of UV-ROS-COX 2- PGE 2 in pterygium, and the effect of COX 2 inhibitors and NSAIDs in preventing primary or recurrent pterygium.
